# Application of Raman Spectroscopy Coupled With Chemometrics for the Detection and Quantification of Mancozeb Residues in Collard Green

**DOI:** 10.1002/ansa.70045

**Published:** 2025-09-14

**Authors:** Saaya Abel Kanai, Wilson Ombati, Robinson Ndegwa, Jared Ombiro Gwaro

**Affiliations:** ^1^ Mathematics and Physical Sciences Maasai Mara University Narok Kenya; ^2^ Department of Metrology Kenya Bureau of Standards Nairobi Kenya; ^3^ Department of Physics The University of Nairobi Nairobi Kenya

**Keywords:** artificial neural networks, convolutional neural networks, principal component analysis, Raman spectroscopy, random forest, support vector machine

## Abstract

The presence of pesticide residues in food crops poses serious health concerns, necessitating precise, rapid and accessible detection techniques. This study investigates the use of Raman spectroscopy combined with advanced data analysis techniques to detect and quantify Mancozeb residues in collard greens. The primary objective was to evaluate the viability of this approach for accurate pesticide residue monitoring in leafy vegetables. Raman spectral data were collected and preprocessed using a standard normalization technique to reduce spectral noise and enhance signal quality. Dimensionality reduction was achieved through a statistical method that extracted key spectral features and successfully differentiated control from treated samples, explaining a combined variance of 86% across the first two principal components. Graphical score plots revealed clear clustering patterns across various residue concentrations, ranging from 0.01 to 0.5 parts per million, with samples categorized according to regulatory residue limits. To further assess predictive capability, several machine learning models were developed for classification and quantification, including deep learning–based and ensemble‐based approaches. Among these, the support vector model achieved the highest classification precision of 95% and demonstrated strong calibration and prediction accuracy. A convolutional neural network achieved 99% training accuracy and 98% testing accuracy, effectively recognizing complex spectral patterns. Statistical validation using analysis of variance confirmed that the observed model differences were significant, supporting the robustness of the selected algorithms. The proposed method accurately quantified Mancozeb residues within the tested range and demonstrated high sensitivity even at low concentration levels. This study highlights the potential of Raman spectroscopy, integrated with computational modelling, as a non‐destructive, fast and cost‐effective tool for pesticide residue detection in food safety applications.

## 1 Introduction

Pesticides are essential in modern agriculture, as they help control pests, weeds and diseases, which improves crop production and contributes to food security [1]. Among the pesticide classes, Mancozeb, a popular dithiocarbamate fungicide, is commonly used due to its broad antifungal properties and affordability [2]. Nevertheless, rising concerns about the excessive or improper use of pesticides have highlighted their potential harm to both human health and the environment [2]. Mancozeb, in particular, can degrade into ethylene thiourea (ETU), a compound known to exhibit carcinogenic, teratogenic and endocrine‐disrupting properties [3]. Mancozeb residues left on harvested crops may contaminate the food chain or surrounding environments, prompting significant environmental and health worries [4]. Although regulatory bodies have established maximum allowable residue levels in food to maintain safety standards, routine monitoring, especially in developing countries, has often been constrained by a lack of accessible and efficient analytical methods [5]. This created a critical need for rapid, accurate and field‐deployable detection methods capable of screening fresh produce for pesticide contamination [6]. Collard greens (*Brassica oleracea* var. acephala), a staple leafy vegetable consumed widely in many regions, have been particularly susceptible to fungal infections and therefore are commonly treated with fungicides like Mancozeb [7]. GC–MS and HPLC, commonly used for pesticide residue detection, are known for their excellent sensitivity and precision [8, 9]. However, they were also time‐consuming, labour‐intensive and reliant on expensive instruments and skilled personnel [10]. These challenges reduce their feasibility for routine use, particularly in field or local market settings. One such alternative has been Raman spectroscopy, a vibrational spectroscopic technique that offers numerous advantages, including non‐destructive testing, minimal sample preparation and the potential for rapid, in‐field analysis [11, 12]. Raman spectroscopy detects specific molecular vibrations that act like spectral fingerprints, allowing for compound identification and measurement [13]. Nonetheless, the interpretation of raw Raman spectra has remained challenging due to background fluorescence, spectral overlap and matrix interference, particularly in complex biological samples like vegetables [14]. To overcome these limitations, Raman spectroscopy is increasingly being integrated with chemometric techniques to improve data interpretation and analytical accuracy [15]. Machine learning techniques, particularly those combining statistical and computational modelling, have shown potential to extract meaningful features from noisy spectral data. Principal component analysis (PCA), for example, is commonly used to reduce the dimension of the data and extract significant spectral characteristics [16, 17, 18]. Although Raman spectroscopy combined with machine learning has shown promising results for pesticide detection, many existing studies have primarily relied on surface‐enhanced Raman spectroscopy (SERS) due to its superior sensitivity [19, 20, 21]. However, SERS‐based techniques often involve costly nanoparticle synthesis, labour‐intensive preparation and poor reproducibility, factors that have limited their scalability in practical, real‐world monitoring [22].

Recent studies on pesticide residue detection using Raman spectroscopy and machine learning have demonstrated promising results, but many are limited by the sample matrices they investigated. For instance, Ma et al. focused on fruits with relatively smooth and uniform surfaces, which present fewer challenges in Raman signal acquisition [23]. However, leafy vegetables such as collard greens have more irregular surface structures and complex cellular anatomy, leading to increased light scattering, baseline variability and matrix‐specific interference. These characteristics make it more difficult to extract consistent and reliable spectral information. The absence of validation on such complex matrices raises concerns about the direct transferability of models trained on simpler substrates. Wang et al. [24] explored deep learning techniques for pesticide quantification using spectral data, but their work was confined to a limited residue concentration range. Such a narrow scope restricts the model's applicability under real‐world agricultural conditions, where residue levels may vary across and beyond regulatory thresholds. Moreover, their study did not benchmark the performance of deep learning algorithms against classical chemometric methods such as partial least squares regression or support vector machines (SVMs). Without comparative evaluation, it remains unclear whether the observed performance improvements were due to the choice of model architecture or the inherent structure of the dataset [24]. Similarly, although Albuquerque and Poppi [25] demonstrated the use of multivariate statistical analysis for pesticide residue detection in agricultural products, their study relied exclusively on linear chemometric techniques such as PCA and partial least squares regression. These methods, although widely used, often fall short in capturing the intricate, non‐linear spectral relationships characteristic of heterogeneous biological samples. Importantly, Albuquerque and Poppi [25] did not explore the application of non‐linear models such as artificial neural networks (ANNs) or convolutional architectures, which are now recognized for their superior pattern recognition capabilities in complex datasets. In contrast, the current study addresses these methodological gaps by targeting collard greens, a matrix known for high pesticide exposure risk and complex spectral features, across a wider concentration range (0.01–0.5 parts per million). We integrate both linear and non‐linear models and systematically compare their performance using rigorous statistical validation. This approach enhances model robustness and accuracy while offering a scalable, non‐destructive solution for pesticide residue quantification in complex leafy vegetable matrices.

Conventional Raman spectroscopy without SERS was employed, reducing cost and preparation steps, while integrating machine learning algorithms across the full Raman spectrum to detect Mancozeb residues in collard greens, a high‐risk leafy matrix not previously emphasized in the literature. In addition, we employed machine learning algorithms, such as SVMs, random forests (RFs), ANNs and convolutional neural networks (CNNs), to develop predictive models for residue classification and quantification. These combined approaches improved detection sensitivity and specificity, even in complex matrices. Table 1 summarizes selected prior studies that used Raman‐based methods for pesticide detection, highlighting their techniques, target compounds, matrices and limitations. This comparison underscores how our methodology advanced the field by eliminating dependency on substrate enhancement and extending detection to underrepresented pesticide–matrix combinations.

**TABLE 1:** Overview of selected Raman‐based studies for pesticide detection.

Technique used	Pesticide detected	Sample matrix	Key limitations	Current study advancement	References
SERS + PCA	Chlorpyrifos	Tomato surface	Dependent on SERS substrates; limited to one pesticide	Applies the entire Raman range with machine learning for leafy vegetables	Ma et al. [23]
SERS + Quenchers	Chlorpyrifos	Citrus fruits	Laborious preparation; affected by background fluorescence	No extraction needed; portable detection	Wang et al. [24]
SERS + MCR	Malathion	Tomato/Plum peels	Restricted to specific matrices; issues with result consistency	Enhances model adaptability using diverse ML algorithms	Albuquerque and Poppi [25]

Abbreviations: CNN, convolutional neural network; MRL, maximum residue limit; PCA, principal component analysis; RMSEC, calibration root mean square error; SERS, surface‐enhanced Raman spectroscopy; SNV, standard normal variate; SVM, support vector machine.

## 2 Experimental Procedures

### 2.1 Chemicals and Materials

Collard greens (*B. oleracea* var. acephala) were collected from five major open‐air markets in Nairobi: Kariokor, Kawangware, Kileleshwa, Gikomba and Ngara. A total of 41 samples were obtained from each market, with purchases made from multiple vendors to capture natural variation and avoid vendor‐specific bias. Samples were taken to the laboratory in clean, labelled polyethylene bags and analysed within 24 h of collection. Mancozeb (ethylene bisdithiocarbamate; EBDC) of analytical grade was used as the target pesticide. Distilled water was employed for all dilutions and solution preparations. Pesticide‐free collard green leaves were included as negative controls. A certified polystyrene standard was utilized for spectrometer calibration and quality assurance of the Raman shifts.

### 2.2 Sample Preparation

In the laboratory, collard green samples were inspected to ensure freshness and absence of visible pesticide residues. Each sample was gently washed with distilled water to remove soil particles and surface contaminants, then air‐dried for 15 min on absorbent paper towels. For pesticide treatments, a 1000 ppm Mancozeb stock solution was prepared by dissolving 9 g of the fungicide in 100 mL of distilled water under continuous stirring until complete dissolution. Working solutions of 0.01, 0.02, 0.1, 0.2, 0.25, 0.3, 0.4 and 0.5 ppm were subsequently obtained through serial dilution using the relation *C*_1_ *V*_1_ = *C*_2_ *V*_2_. *C*_1_ represented the initial concentration of the stock solution *V*_1_, the volume of the stock solution added, *C*_2_ was the final concentration after dilution, and *V*_2_ was the volume of the diluent added. The selected concentration range was designed to reflect residue levels relevant to food safety monitoring. Treatments included concentrations below the maximum residue limit (MRL), at the MRL and above the MRL (**3 ppm**) to provide a comprehensive evaluation of Raman spectroscopy performance across regulatory thresholds. This ensured that the experimental design captured realistic contamination scenarios likely to be encountered in collard greens [26]. Each working solution (2 mL) was uniformly sprayed onto collard green leaves using a micropipette‐tipped hand sprayer held approximately 15 cm above the leaf surface to ensure even distribution. The sprayed leaves were placed on sterile glass plates and allowed to dry at room temperature (25°C ± 2°C) for 1 h to mimic typical post‐harvest handling and environmental exposure. Control samples were prepared by spraying pesticide‐free leaves with 2 mL of distilled water. To account for sample heterogeneity, 41 spectra were collected from random points on each leaf. This was repeated for three biological replicates per concentration level, with each replicate derived from an independently treated leaf. Thus, the total dataset consisted of spectra from multiple concentrations, replicates and sampling locations to enhance statistical robustness.

### 2.3 Instrumentation and Settings

Raman spectra were acquired using a dispersive Raman spectrometer (Airix Corporation) equipped with 600, 1200 and 1800 lines/mm diffraction gratings and two excitation lasers (785 and 532 nm; Princeton Instruments). The 785 nm laser was selected to minimize background fluorescence and photodegradation of the plant matrix. Instrument calibration was performed daily using a certified polystyrene standard to verify wavenumber accuracy and signal reproducibility. Spectral acquisition was carried out using the 600 lines/mm grating centred at 1000 cm^−1^, covering the 200–2000 cm^−1^ fingerprint region. A ×10 objective lens (NA = 0.3) provided a beam spot size of approximately 68.5 µm. Instrument settings included a 10‐s exposure time, 10 accumulations per point and a laser power of ∼9.1 mW (50% of the maximum 18.2 mW). The instrument was housed in a temperature‐controlled laboratory (22°C ± 1°C), and measurements were performed in a dimly lit room to reduce interference from ambient light. All spectra were preprocessed prior to chemometric analysis. Standard normal variate (SNV) transformation was applied to correct baseline shifts and normalize intensity variations. Additional preprocessing steps included cosmic ray removal and smoothing using a Savitzky–Golay filter to improve the signal‐to‐noise ratio [27]. These preprocessing steps ensured that spectral differences reflected pesticide residue variations rather than instrumental or environmental artefacts.

## 3 Results and Discussion

### 3.1 Preprocessing and PCA of Raman Spectra

**FIGURE 1:**
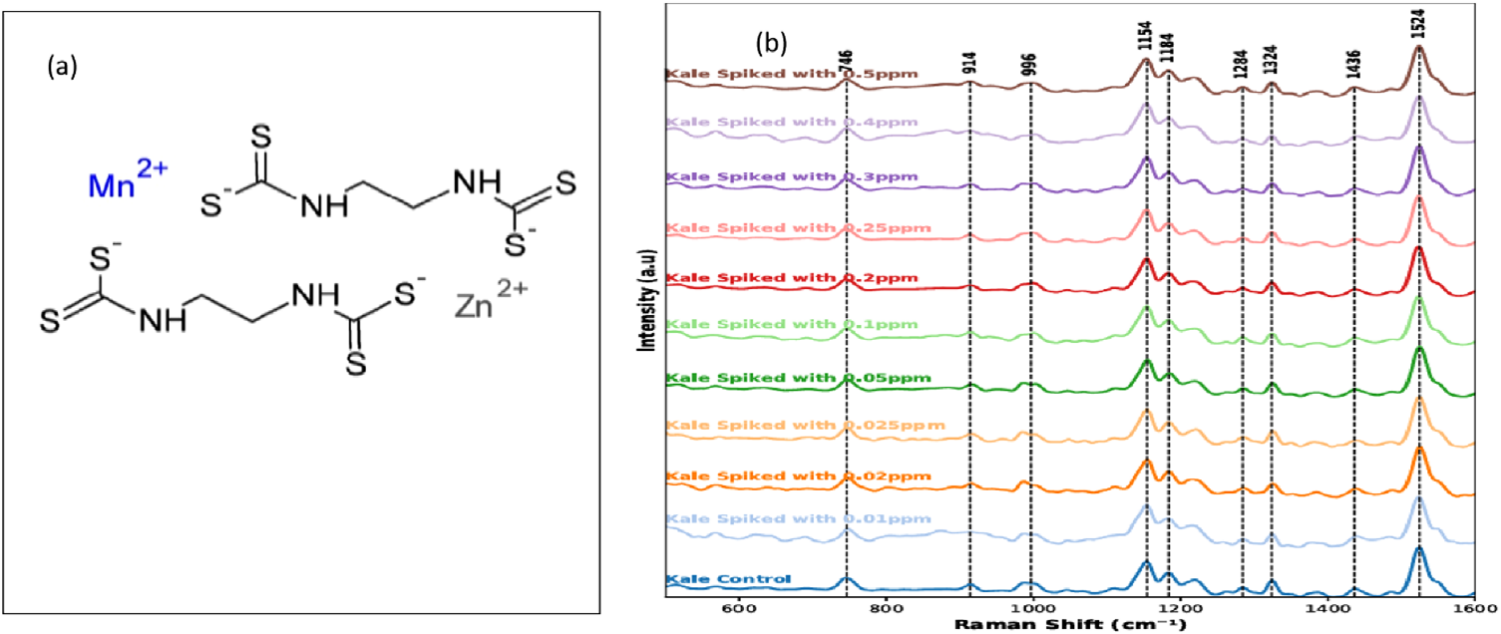
(a) Molecular structure of Mancozeb, showing two ethylene bisdithiocarbamate (EBDC) ligands coordinated with manganese (Mn^2+^) and zinc (Zn^2+^) ions through sulphur donor atoms. (b) Raman spectral profiles of collard green samples spiked with varying concentrations of Mancozeb, highlighting progressive changes in peak intensity and sharpness within the fingerprint region (600–1600 cm^−1^). Key characteristic peaks correspond to C–S, N–H bending and C–N and C–C skeletal vibrations, with notable bands at 1154 cm^−1^ (symmetric C–N stretching of dithiocarbamates) and 1324 cm^−1^ (CH_2_ bending and aliphatic chain deformations).

**FIGURE 2:**
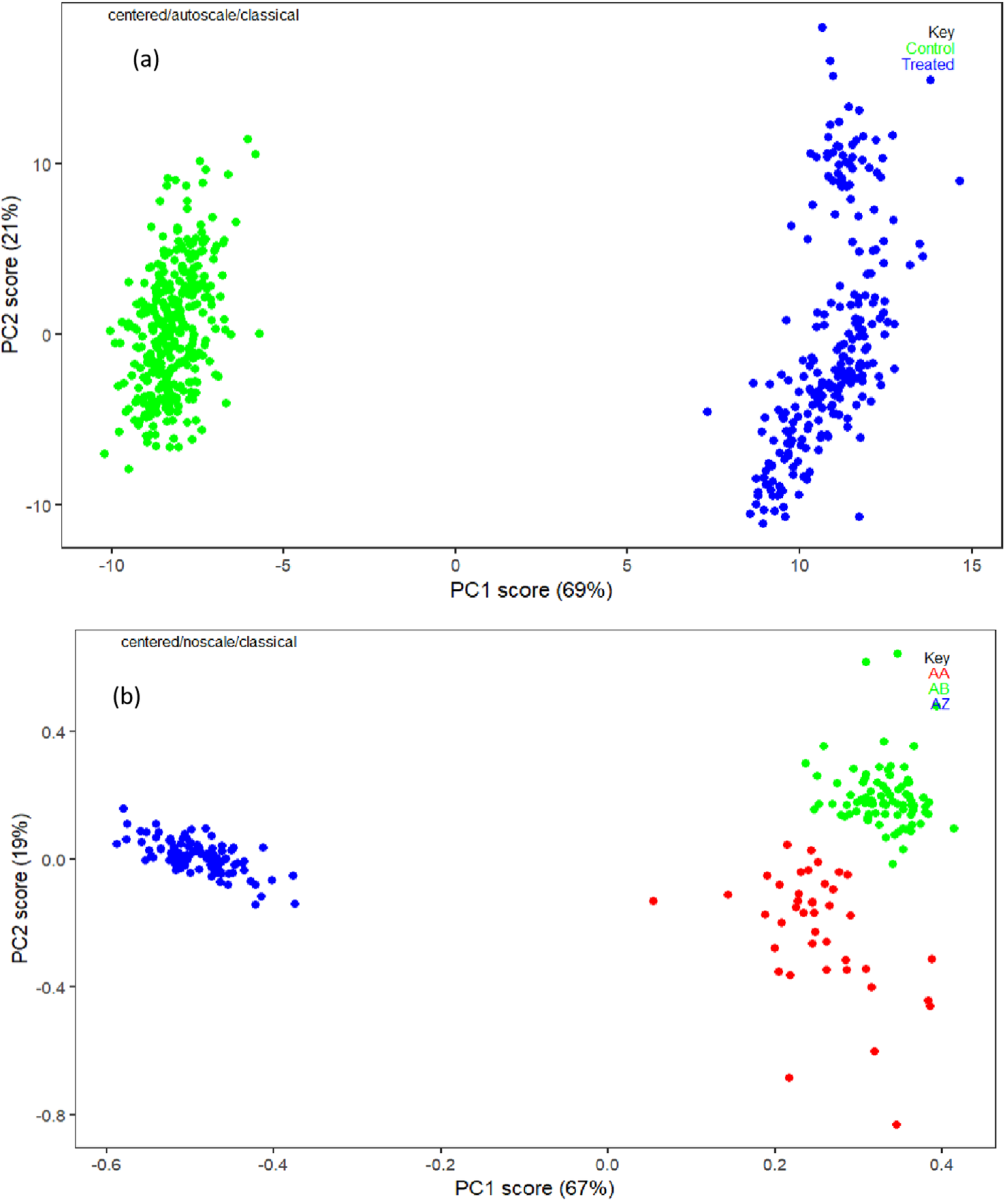
(a) PCA score plot showing the separation between control (0 ppm) and Mancozeb‐treated (0.1–0.5 ppm) collard green samples based on Raman spectra (500–1600 cm^−1^), with PC1 and PC2 accounting for 69% and 21% of the total variance, respectively. (b) PCA score plot further classifying samples into three distinct groups: control (0 ppm, labelled AZ), below MRL (0.1–0.3 ppm, labelled AA) and above MRL (0.4–0.5 ppm, labelled AB), with PC1 and PC2 explaining 67% and 19% of the variance. A progressive increase in concentration is observed along the PC1 axis. PC, principal component.

Figure 1a depicts the molecular structure of Mancozeb, a coordination complex fungicide composed of two EBDC ligands chelated with manganese (Mn^2+^) and zinc (Zn^2+^) ions. Each EBDC ligand contains a central ethylene backbone flanked by thiocarbamate functional groups, with sulphur atoms serving as key donor sites for metal coordination. The Mn^2+^ and Zn^2+^ centres are stabilized by four sulphur donor atoms each, forming stable chelate rings that enhance the compound's fungicidal activity and structural stability. The abundance of sulphur atoms contributes to distinct Raman spectral signatures, particularly in the fingerprint region. As shown in Figure 1b, the Raman spectral profiles of collard green samples spiked with varying concentrations of Mancozeb exhibit progressive changes in peak intensity and sharpness along the fingerprint region (600–1600 cm^−1^). Characteristic peaks were observed at approximately 746, 914, 996, 1154, 1184, 1284, 1324, 1436 and 1524 cm^−1^, corresponding to vibrational modes associated with C–S, N–H bending and C–N, C–C skeletal stretches. The band near 1154 cm^−1^ is attributed to symmetric C–N stretching typical of dithiocarbamate moieties, whereas the peak at 1324 cm^−1^ aligns with CH_2_ bending and aliphatic chain deformations. The molecular structure of Mancozeb (Figure 1a) provides the chemical basis for the characteristic Raman bands observed in the spiked kale spectra (Figure 1b). These spectral features, linked to functional groups within the molecule, are further explored in Figure 2, which illustrates the spectral variations after preprocessing and the resulting classification performance of the applied chemometric models.

To segregate the Raman spectral data across different concentrations, we applied PCA. A total of 41 spectra were collected for each of the 5 selected concentrations, used for both qualitative and quantitative modelling. Rather than averaging, all 41 spectra per concentration level were treated as individual input samples in both PCA and machine learning analysis. This approach allowed the model to capture the inherent within‐sample variance and provided a larger dataset for training, improving statistical robustness. To manage spectral variability, SNV preprocessing was applied to normalize intensity differences across spectra. PCA was then employed not only to reduce dimensionality but also to retain variance structure, with principal components (PCs) representing the shared spectral patterns among replicates. As a result, variability across measurements was not suppressed but rather incorporated into the model structure, aiding in the discrimination of pesticide concentration levels. Figure 2 shows the results of PCA applied to the Raman spectra, highlighting distinct separation among control, below‐MRL and above‐MRL groups.

The resulting PCA score plots demonstrate distinct clustering based on Mancozeb concentration levels. The dataset was divided into control samples (0 ppm) and treated samples ranging from 0.1 to 0.5 ppm. These were further categorized into two subgroups: below the MRL (0.1–0.3 ppm) and above the MRL (0.4–0.5 ppm). The PCA analysis, performed over the spectral fingerprint region (500–1600 cm^−1^), enabled effective visualization of concentration‐dependent separation.

**FIGURE 3:**
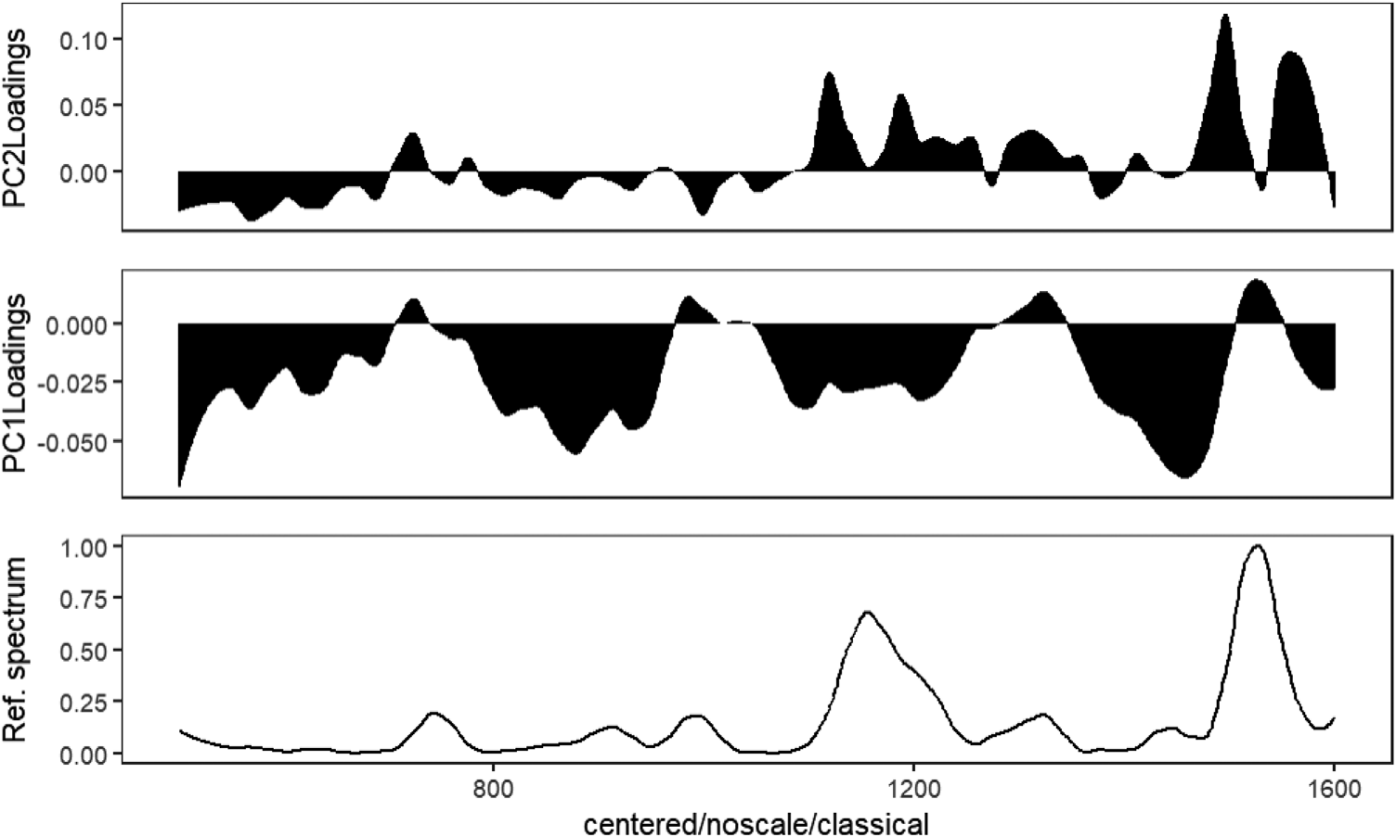
PC1 and PC2 loading plots showing spectral regions contributing to sample separation. Positive PC1 is dominated by peaks around 1550 cm^−1^, whereas negative PC1 aligns with features between 1300 and 1500 cm^−1^. Positive PC2 highlights spectral activity within 1200–1600 cm^−1^, and negative PC2 corresponds to 500–800 cm^−1^. The accompanying reference Raman spectrum aids in interpreting these vibrational modes, supporting Mancozeb detection. PC, principal component.

PC1 accounted for 67%–69% of the total spectral variance, whereas PC2 explained 19%–21%, depending on the subgrouping. The score plots revealed clear groupings, with control samples (AZ) primarily occupying the negative PC1 space, below‐MRL samples (AA) clustering around the centre, and above‐MRL samples (AB) shifting towards the positive PC1 axis. This concentration‐based trend supports the discriminatory power of PCA for detecting pesticide residue levels in collard greens. To provide further insights into this grouping, the loading plot shown in Figure 3 was employed for interpretation. PC1, which accounts for 69% and 67% of the variance, respectively, is associated with a prominent wavelength at 1550 cm^−1^ on the positive axis, whereas the negative PC1 loadings correspond to spectral features in the 1300–1500 cm^−1^ region. Similarly, PC2, which explains 21% and 19% of the overall variance, is linked to vibrational modes in the 1200–1600 cm^−1^ range (positive PC2) and 500–800 cm^−1^ (negative PC2), as indicated in the same figure.

### 3.2 Spectra Data Analysis

In this study, various preprocessing techniques were applied to the acquired Raman spectra, including SNV, first derivative and second derivative transformations. These methods are widely used to enhance spectral resolution and mitigate challenges such as overlapping peaks and baseline variations. The SNV transformation was applied to each spectrum to normalize intensity and correct for scattering effects arising from particle size variation and sample heterogeneity. For a given original spectrum $x=[x_{1},x_{2},\text{…},x_{N}]$ with *N* wavelength points, the mean of the spectrum is given by $\mu =\frac{1}{N}\sum_{i=1}^{N}x_{i}$ at ith wavelength λ and the standard deviation of the spectrum is given by $\sigma =\sqrt{\frac{1}{N-1}}\sum_{i=1}^{N}{\left(x_{i}-\mu \right)}^{2}$, then SNV transformed spectrum is defined as $x_{\mathrm{SNV}}$:

(1) $$x_{\mathrm{SNV}}=\frac{x_{i}-\mu }{\sigma }$$

This transformation reduced high‐frequency noise, corrected baseline drift and improved comparability across samples. To further resolve subtle spectral features, the first derivative of the SNV; corrected spectra was calculated using the Savitzky–Golay algorithm:

(2) $$x_{\mathrm{SNV}}^{\prime }=\frac{d}{d\lambda }x_{\mathrm{SNV}}$$

This enhanced peak resolution and corrected baseline shifts, making minor features more distinguishable. However, it also amplified random noise in some regions of the spectra. The second derivative transformation was also applied to the SNV‐corrected spectra:

(3) $$x_{\mathrm{SNV}}^{\prime \prime }=\frac{d^{2}}{d\lambda^{2}}x_{\mathrm{SNV}}^{\prime }$$

This method further improved resolution of overlapping peaks and sharpened spectral features but, as with the first derivative, increased noise sensitivity. Although all three methods revealed clear distinctions among collard green samples with varying Mancozeb concentrations, the differences were most apparent in band intensities and sharpness along the fingerprint region (600–1600 cm^−1^). SNV primarily standardized the baseline and intensity levels, the first derivative highlighted subtle peak position shifts, and the second derivative enhanced the separation of closely spaced peaks. These observations are consistent with previous findings by Nitze et al. [28] and Wang et al. [24], which reported that the effectiveness of machine learning models for pesticide detection is strongly influenced by the choice of spectral preprocessing. In our case, SNV provided the most stable baseline correction, whereas derivative‐based approaches offered better resolution for complex spectral regions at the cost of increased noise.

### 3.3 Selection of Variables in Multivariate Modelling of Raman Spectra

**FIGURE 4:**
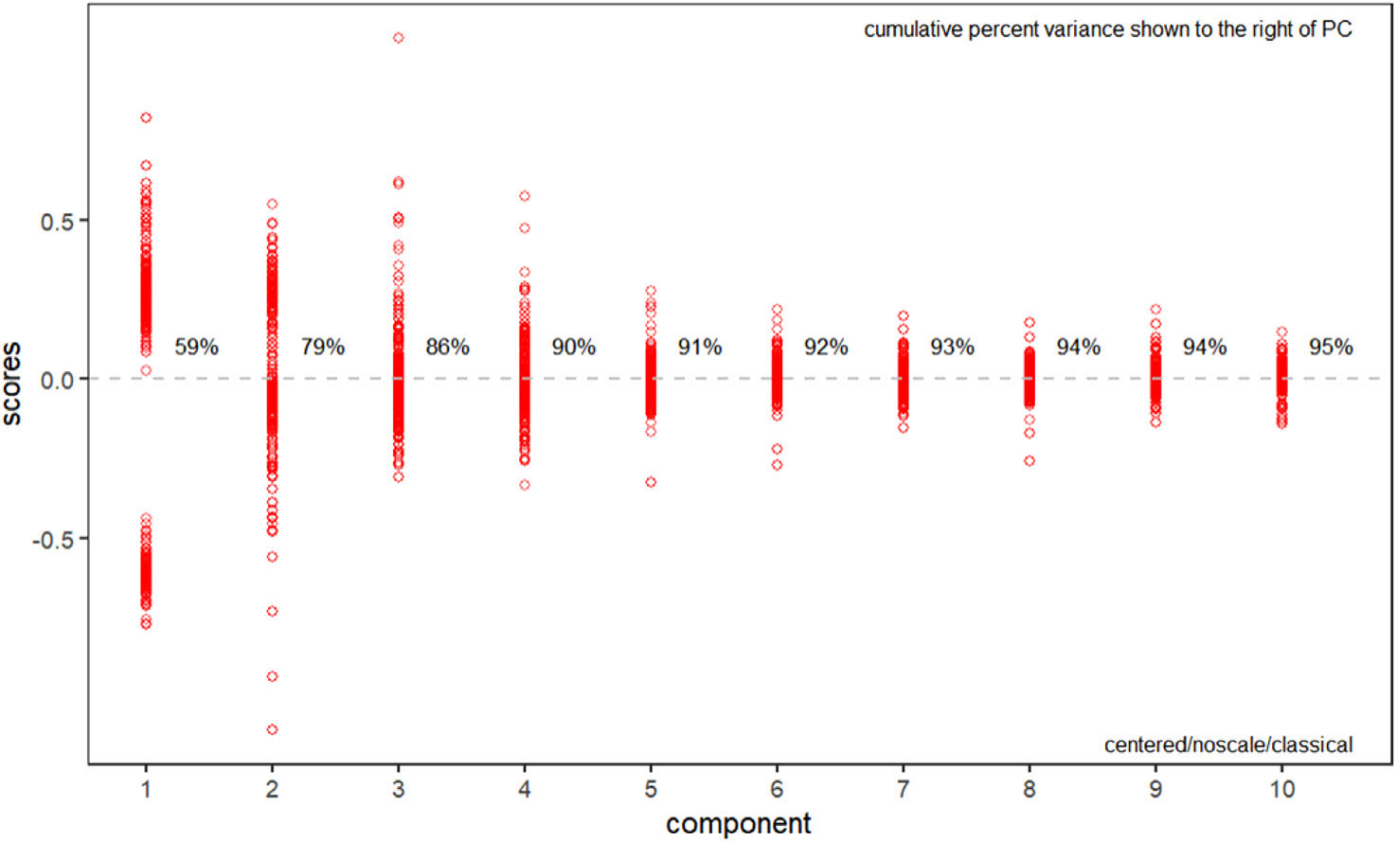
Key components displayed in a PCA scree plot. Four principal components were selected as they accounted for over 99% of the cumulative variance. PC, principal component.

When the entire spectral data was used for multivariate calibration, it led to machine learning models with limited predictive performance and overall reliability. Therefore, only the most informative variables were selected for the model. To achieve this, PCA was applied to eliminate unnecessary features and noise. The first four PCs were derived from the result matrix and used in the machine learning models. The appropriate number of PCs to include was determined by the scree plot 10. The first four PCs were retained, as they provided an optimal balance between reducing dimensionality and preserving meaningful spectral information. As shown in the scree plot in Figure 4, these four PCs together accounted for over 99% of the total variance, thereby satisfying our predefined cumulative variance threshold. Each additional component beyond the fourth contributed less than 2% of incremental variance and exhibited eigenvalues below one, indicating limited explanatory value. This informed selection helped to simplify the model, reduce the risk of overfitting and maintain the most discriminative spectral features essential for accurate classification and regression performance.

### 3.4 Multivariate Analysis of Raman Spectra

The relationship between pesticide concentration and PC data was evaluated using SVM and RF algorithms focused on Mancozeb. These models aimed to estimate pesticide concentrations that were generated through controlled experimental dilutions. Model predictions showed a strong resemblance to the laboratory‐prepared reference concentrations. Evaluation of the calibration models’ accuracy was performed using RMSE and *R* ^2^ as performance indicators. The purpose of developing both models was to determine which exhibited superior performance during training and testing phases [29]. SVM maintained consistent accuracy on both training and testing datasets regardless of the preprocessing technique applied. Notably, the combination of SNV preprocessing with the 0th‐order derivative yielded superior results, with a calibration root mean square error (RMSEC) of 0.045 and a prediction root mean square error (RMSEP) of 0.051. These results suggest that SVM is a robust model with strong generalization capabilities for unfamiliar datasets. On the other hand, RF showed a marked difference between its training and testing performance. SVM's robustness can be attributed to its margin maximization, suitability for high‐dimensional and limited datasets, use of kernel functions for non‐linearities and built‐in regularization. According to the no free lunch theorem, no single model is universally superior across all types of problems [30]. The specific characteristics of the problem at hand influence the effectiveness of any given model. This implies that the differences between these two models extend beyond their mathematical structures; they also differ in the way they learn from the dataset. Consequently, these models can be seen as complementary; each model offers unique strengths, depending on the characteristics of the data and the specific task at hand.

**FIGURE 5:**
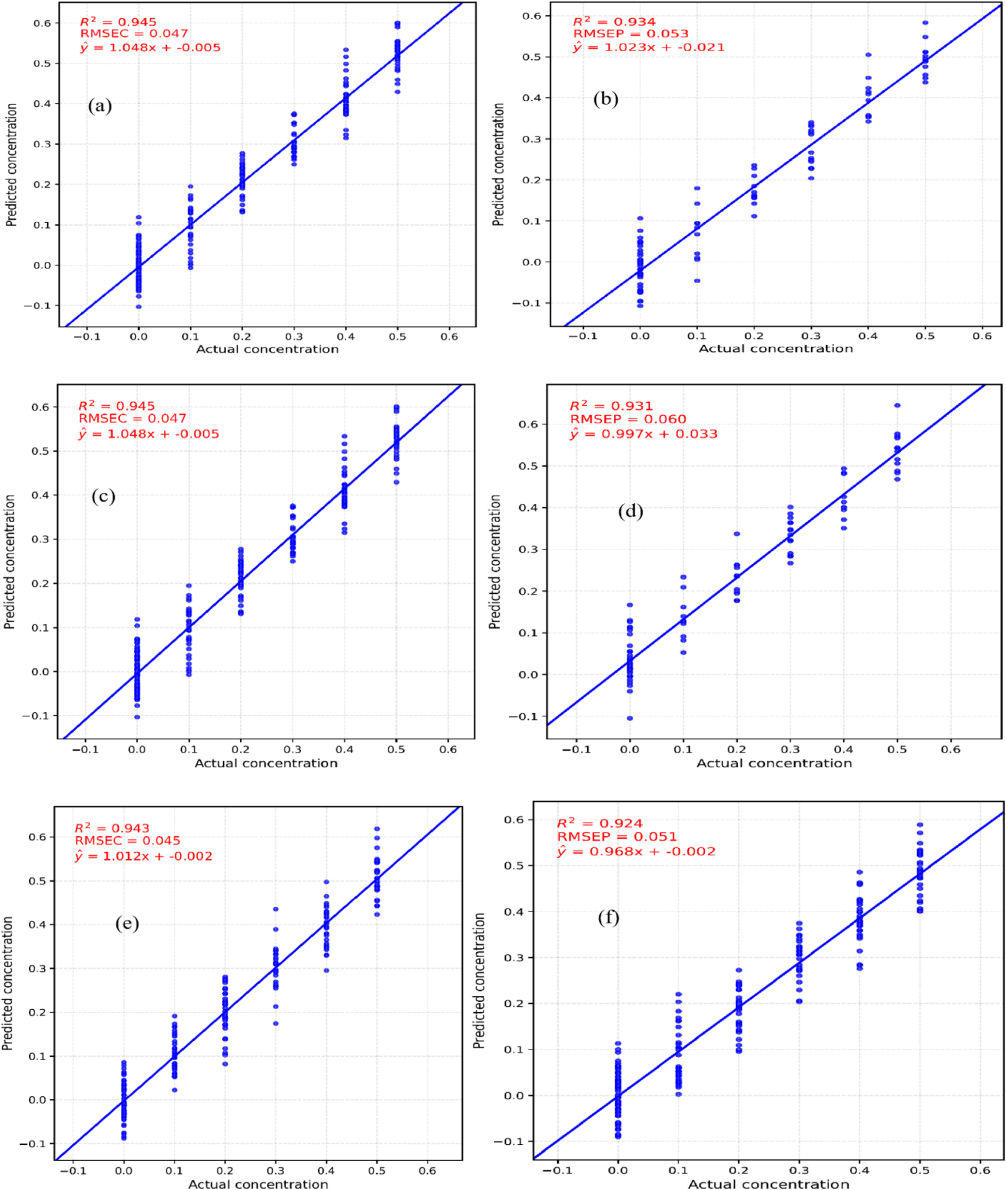
(a) Correlation plot of actual versus predicted Mancozeb residues for the training set using SNV first derivative SVM model, (b) correlation plot of actual versus predicted Mancozeb residues for the testing set using SNV first derivative SVM model, (c) correlation plot of actual versus predicted Mancozeb residues for the training set using SNV second derivative SVM model, (d) correlation plot of actual versus predicted Mancozeb residues for the testing set using SNV second derivative SVM model, (e) correlation plot of actual versus predicted Mancozeb residues for the training set using SNV SVM model, (f) correlation plot of actual versus predicted Mancozeb residues for the testing set using SNV SVM model. RMSEC, calibration root mean square error; RMSEP, prediction root mean square error.

To evaluate the SVM model's performance, correlation plots were generated to compare the predicted and actual concentrations of Mancozeb residues in both training and testing sets. These plots are presented in Figure 5 to illustrate how well the model performed under different preprocessing methods.

**FIGURE 6:**
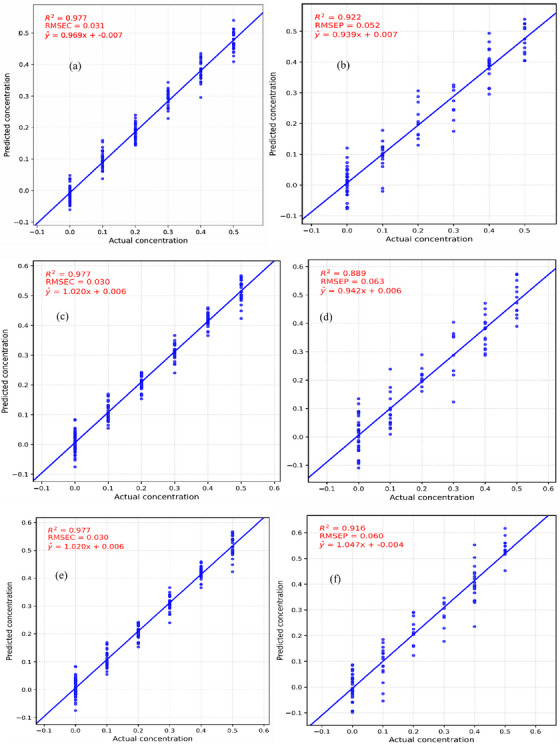
(a) Correlation plot of actual versus predicted Mancozeb residues for the training set using SNV first derivative RF model, (b) correlation plot of actual versus predicted Mancozeb residues for the testing set using SNV first derivative RF model, (c) correlation plot of actual versus predicted Mancozeb residues for the training set using SNV second derivative RF model, (d) correlation plot of actual versus predicted Mancozeb residues for the testing set using SNV second derivative RF model, (e) correlation plot of actual versus predicted Mancozeb residues for the training set using SNV RF model, (f) correlation plot of actual versus predicted Mancozeb residues for the testing set using SNV RF model. RMSEC, calibration root mean square error; RMSEP, prediction root mean square error.

The RF model's effectiveness in predicting Mancozeb residue levels was also analysed using correlation plots for both the training and testing data. Figure 6 illustrates how the model performed across various preprocessing approaches.

The performance metrics for SVM and RF models under different preprocessing techniques are summarized in Table 2.

**TABLE 2:** Model development of support vector machine (SVM) and random forest (RF).

Quantitative models	Preprocessing methods	RMSEC (ppm)	RMSEP (ppm)	*R* ^2^ training	*R* ^2^ testing
SVM	SNV	0.045	0.051	0.943	0.924
	1st derivative	0.047	0.053	0.945	0.934
	2nd derivative	0.047	0.060	0.945	0.931
RF	SNV	0.030	0.060	0.977	0.916
	1st derivative	0.031	0.052	0.977	0.922
	2nd derivative	0.030	0.063	0.977	0.889

Abbreviations: RF, random forest; RMSEC, calibration root mean square error; RMSEP, prediction root mean square error; SNV, standard normal variate; SVM, support vector machine.

### 3.5 Classification of Mancozeb Contaminated Collard Green Samples

**FIGURE 7:**
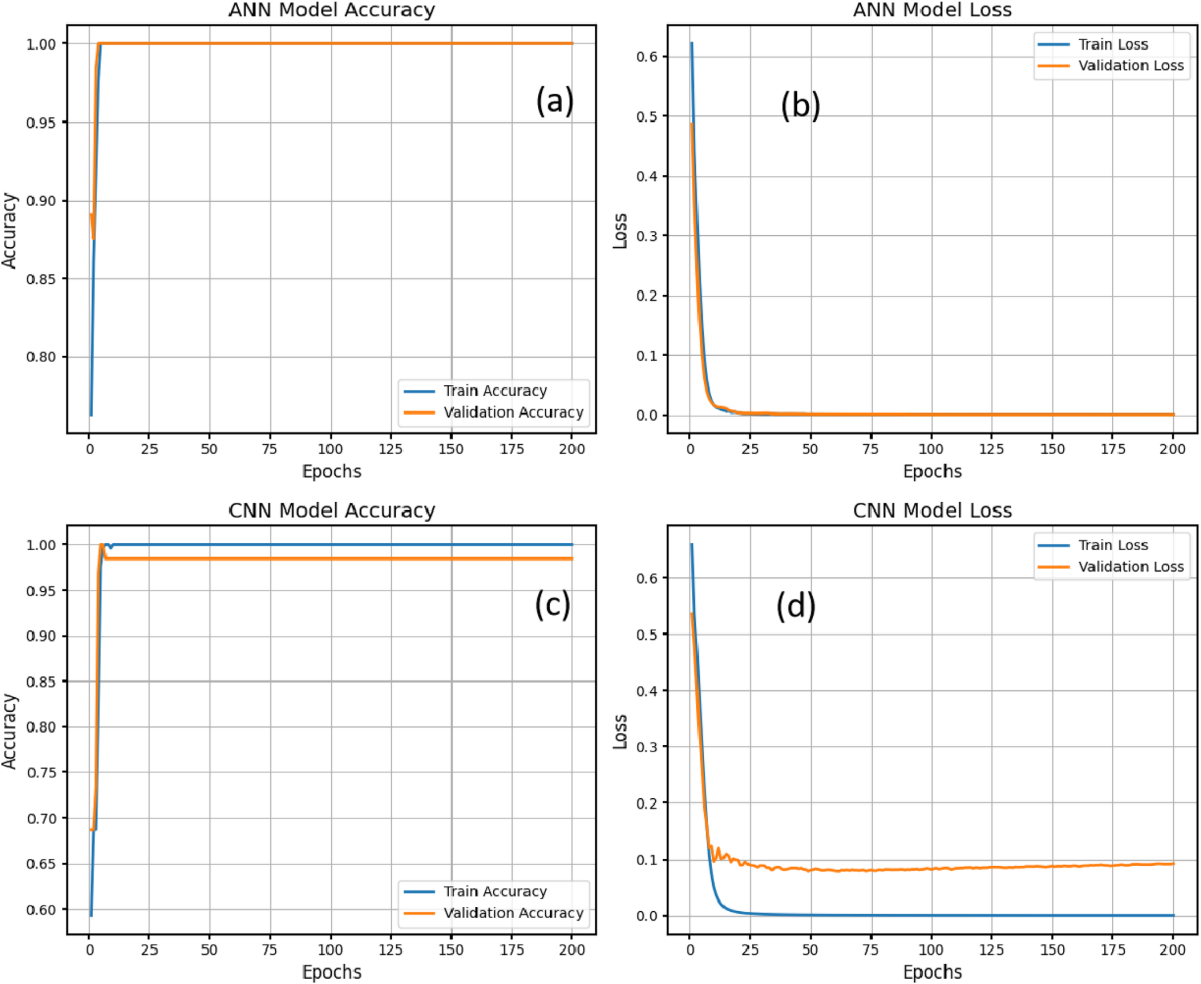
Training performance of classification models for detecting Mancozeb contamination in collard greens: (a) ANN accuracy curve, (b) ANN loss curve, (c) CNN accuracy curve and (d) CNN loss curve. CNN achieved higher testing accuracy on raw spectral data (98%) compared to PCA‐processed data (96%), whereas ANN achieved up to 99% accuracy in training and 94%–95% in testing. ANN, artificial neural network; CNN, convolutional neural network.

This section presents the development of classification models to categorize collard green samples into three predefined groups based on Mancozeb residue levels: control, below MRL and above MRL. Two machine learning approaches, ANNs and CNNs, were applied to detect Mancozeb contamination using both raw spectral data and spectra processed with PCA. For the ANN model, classification accuracy during training reached 98% for raw spectra and 94% for PCA‐transformed data. In testing, the model achieved 99% accuracy on raw data but slightly lower performance of 95% on PCA‐processed data. This drop in accuracy on PCA data suggests that dimensionality reduction, although helpful in reducing computational complexity, may also eliminate subtle but relevant spectral features. The CNN model demonstrated training accuracies of 99% with raw spectra and 98% with PCA‐transformed spectra. During testing, it achieved 98% accuracy with raw data and 96% with PCA data. The consistently high performance of CNN can be attributed to its ability to capture local spectral features and model complex patterns through convolutional and pooling layers. These layers help to reduce overfitting and improve the network's generalization ability, even without data augmentation. The fact that CNN achieved its highest testing accuracy on raw spectra (98%) underscores its strength in learning directly from high‐dimensional inputs, without the need for PCA feature reduction. To ensure robustness and minimize overfitting, the CNN was trained using an 80/20 train‐validation split, L2 regularization (*λ* = 0.02) and a dropout rate of 30% after dense and pooling layers. Model validation was performed with an independent dataset excluded from training. Although *k*‐fold cross‐validation was not applied, consistent performance across multiple independent runs confirmed the stability of both ANN and CNN models. In addition, the CNN benefitted from early stopping, L2 penalties and aggressive dropout, which together ensured effective generalization beyond the training set. The 20% validation split was kept entirely independent from the training data throughout model tuning and evaluation. Figure 7 presents the training behaviour of the ANN and CNN models. Panel (a) shows the ANN training accuracy curve, whereas panel (b) depicts its corresponding training loss curve. Panel (c) illustrates the CNN training accuracy curve, and panel (d) shows the CNN training loss curve. These plots demonstrate the progressive improvement in classification performance and the concurrent reduction in model error over successive epochs for both neural network architectures.

### 3.6 Statistical Comparison of Model Performance

**FIGURE 8:**
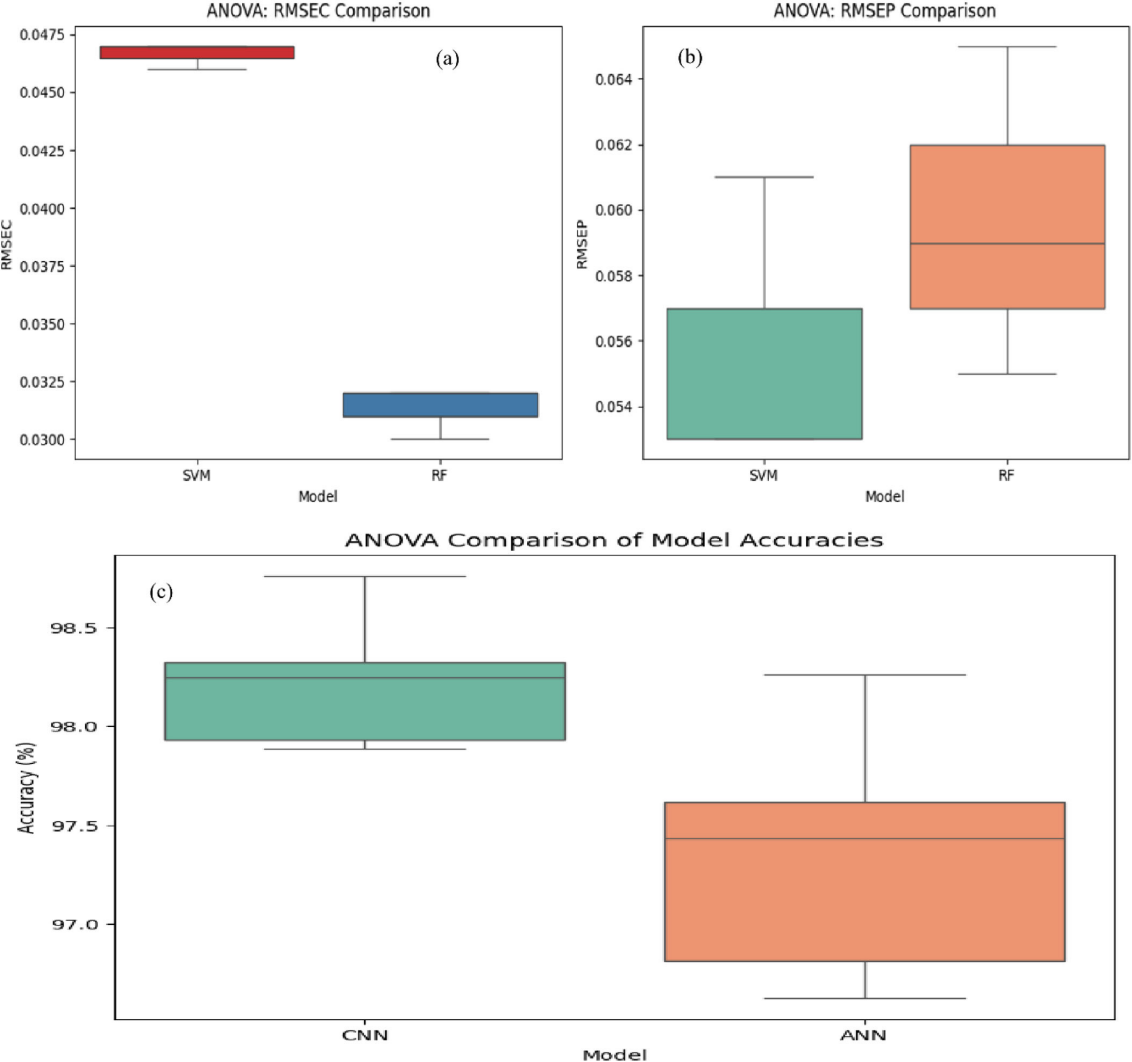
Combined boxplots illustrating model performance under different preprocessing techniques: (a) RMSEC values for SVM and RF models, with SVM showing significantly lower errors (*p* < 0.05), indicating superior calibration; (b) RMSEP values for the same models, with no significant differences observed (*p* > 0.05), indicating comparable predictive ability; (c) Boxplot comparing classification accuracies across CNN and ANN models. CNN showed the highest median accuracy and lowest spread. Statistical analysis via one‐way ANOVA confirmed that these differences were significant (*p* < 0.05). ANN, artificial neural network; ANOVA, analysis of variance; CNN, convolutional neural network; RMSEC, calibration root mean square error; RMSEP, prediction root mean square error.

To statistically assess the performance differences between models and preprocessing techniques, one‐way analysis of variance (ANOVA) was conducted. For regression analysis, ANOVA was applied to RMSEC and RMSEP values obtained from repeated model runs. The results indicated that SVM achieved significantly lower RMSEC values than RF (*p* < 0.05), confirming its superior calibration capability. However, the difference in RMSEP between SVM and RF was not statistically significant (*p* > 0.05), suggesting that both models exhibited comparable predictive accuracy. For classification, ANOVA was performed on the accuracies of ANN and CNN models across repeated runs (*n* = 5). The analysis revealed that CNN achieved the highest median classification accuracy with the smallest variance, followed by ANN, and these differences were statistically significant (*p* < 0.05). Figure 8 summarizes the comparative performance of regression and classification models under different preprocessing techniques.

## 4 Conclusion

This research confirmed the effectiveness of Raman spectroscopy, integrated with chemometric techniques and machine learning models, as a reliable and efficient method for detecting Mancozeb residues in collard greens. The preprocessing of Raman spectra utilizing SNV and PCA significantly reduced spectral noise and improved model performance, with PCA capturing 85% of variance. Among the evaluated models, SVM achieved the highest accuracy of 95%, outperforming other models such as CNN, ANN and RF. The system accurately quantified Mancozeb residues within the tested concentration range of 0.01–0.5 ppm. To support these claims, a statistical analysis using one‐way ANOVA was performed, confirming that the observed differences in model performance, particularly in classification accuracy and calibration error, were statistically significant (*p* < 0.05). This validation strengthens the robustness of the selected models, especially the superior performance of CNN and SVM. The results emphasized the non‐destructive, fast and affordable potential of Raman spectroscopy as a substitute for traditional methods of residue analysis, making it a promising tool for food safety and regulatory compliance. Building on the current findings, future research could focus on adapting this approach for in‐field, real‐time detection using portable Raman devices, enabling routine monitoring in farms and local markets. Further model generalization could be achieved by incorporating additional pesticide types, broader agricultural matrices and seasonal sampling. Moreover, integrating Raman‐based detection with cloud platforms or mobile applications may enhance its accessibility and utility for farmers, food inspectors and regulatory agencies, particularly in resource‐limited settings. These advancements could transform pesticide monitoring into a rapid, scalable and technology‐driven solution, supporting safer agricultural practices and stronger public health outcomes.

## Author Contributions

**Saaya Abel Kanai**: investigation, methodology, visualization, writing – review & editing, writing – original draft, data curation, funding acquisition. **Wilson Ombati**: writing – review & editing, project administration, methodology, supervision. **Robinson Ndegwa**: conceptualization, validation, methodology, resources, project administration, writing – review & editing. **Jared Ombiro Gwaro**: conceptualization, validation, methodology, project administration, supervision, writing – review & editing.

## Disclosure

The funding organization was not involved in designing, collecting data, analysing, interpreting or preparing this manuscript. No additional financial support or competition in economic interests is declared.

## Conflicts of Interest

The authors declare no conflicts of interest.

## Data Availability

The data that support the findings of this study are available from the corresponding author upon reasonable request.

## References

[1] J. Popp , K. Peto˝ , and J. Nagy , “Pesticide Productivity and Food Security. A Review,” Agronomy for Sustainable Development 33 (2013): 243–255.

[2] K. Gyawali , “Pesticide Uses and Its Effects on Public Health and Environment,” Journal of Health Promotion 6 (2018): 28–36.

[3] P. Cocco , “Time for Re‐Evaluating the Human Carcinogenicity of Ethylenedithiocarbamate Fungicides? A Systematic Review,” International Journal of Environmental Research and Public Health 19, no. 5 (2022): 2632. 35270318 10.3390/ijerph19052632 PMC8909994

[4] S. Ali , M. I. Ullah , A. Sajjad , Q. Shakeel , and A. Hussain , “Environmental and Health Effects of Pesticide Residues,” in Sustainable Agriculture Reviews 48: Pesticide Occurrence, Analysis and Remediation (Springer, 2021).

[5] P. Dureja , S. B. Singh , and B. S. Parmar , “Pesticide Maximum Residue Limit (MRL): Background, Indian Scenario,” Pesticide Research Journal 27, no. 1 (2015): 4–22.

[6] T. Mutunga , S. Sinanovic , and C. S. Harrison , “Integrating Wireless Remote Sensing and Sensors for Monitoring Pesticide Pollution in Surface and Groundwater,” Sensors 24, no. 10 (2024): 3191. 38794044 10.3390/s24103191 PMC11125874

[7] B. Jagadeesh , S. Maurya , P. Hemalatha , and A. Lingam , Diseases and Disorders of Cole Crops (Stem Brassicas) and Their Management (Apple Academic Press, 2022).

[8] N. Gao , X. Guo , K. Zhang , and D. Hu , “High‐Performance Liquid Chromatography and Gas Chromatography—Mass Spectrometry Methods for the Determination of Imidacloprid, Chlorpyrifos, and Bifenthrin Residues in Tea Leaves,” Instrumentation Science & Technology 42, no. 3 (2014): 267–277.

[9] S. Sindhu and A. Manickavasagan , “Nondestructive Testing Methods for Pesticide Residue in Food Commodities: A Review,” Comprehensive Reviews in Food Science and Food Safety 22, no. 2 (2023): 1226–1256. 36710657 10.1111/1541-4337.13109

[10] S. Boonyong , S. Hunnangkul , S. Vijit , et al., “High‐Throughput Detection of Parasites and Ova in Stool Using the Fully Automatic Digital Feces Analyzer, Orienter Model fa280,” Parasites & Vectors 17, no. 1 (2024): 13. 38185634 10.1186/s13071-023-06108-1 PMC10771706

[11] J. M. Chalmers , H. G. Edwards , and M. D. Hargreaves , “Vibrational Spectroscopy Techniques: Basics and Instrumentation,” in Infrared and Raman Spectroscopy in Forensic Science (Wiley, 2012).

[12] B. Eckert and R. Steudel , Molecular Spectra of Sulfur Molecules and Solid Sulfur Allotropes (Springer, 2003).

[13] M. O. Amin , E. Al‐Hetlani , and I. K. Lednev , “Detection and Identification of Drug Traces in Latent Fingermarks Using Raman Spectroscopy,” Scientific Reports 12, no. 1 (2022): 3136. 35210525 10.1038/s41598-022-07168-6 PMC8873478

[14] A. Saletnik , B. Saletnik , and C. Puchalski , “Overview of Popular Techniques of Raman Spectroscopy and Their Potential in the Study of Plant Tissues,” Molecules (Basel, Switzerland) 26, no. 6 (2021): 1537. 33799702 10.3390/molecules26061537 PMC7999012

[15] H. P. Wang , P. Chen , J. W. Dai , et al., “Recent Advances of Chemometric Calibration Methods in Modern Spectroscopy: Algorithms, Strategy, and Related Issues,” TrAC Trends in Analytical Chemistry 153 (2022): 116648.

[16] H. Guo , K. J. Marfurt , and J. Liu , “Principal Component Spectral Analysis,” Geophysics 74, no. 4 (2009): P35–P43.

[17] S. Farquharson , C. Brouillette , W. Smith , and C. Shende , “A Surface‐Enhanced Raman Spectral Library of Important Drugs Associated With Point‐of‐Care and Field Applications,” Frontiers in Chemistry 7 (2019): 706. 31709234 10.3389/fchem.2019.00706 PMC6823623

[18] Z. Xia , Y. Chen , and C. Xu , “Multiview PCA: A Methodology of Feature Extraction and Dimension Reduction for High‐Order Data,” IEEE Transactions on Cybernetics 52, no. 10 (2021): 11068–11080. 10.1109/TCYB.2021.3106485 34469326

[19] S. Srivastava , W. Wang , W. Zhou , M. Jin , and P. J. Vikesland , “Machine Learning‐Assisted Surface‐Enhanced Raman Spectroscopy Detection for Environmental Applications: A Review,” Environmental Science & Technology 58, no. 47 (2024): 20830–20848. 39537382 10.1021/acs.est.4c06737 PMC11603787

[20] A. Saletnik , B. Saletnik , G. Zaguła , and C. Puchalski , “Raman Spectroscopy for Plant Disease Detection in Next‐Generation Agriculture,” Sustainability 16, no. 13 (2024): 5474.

[21] C. M. Tsen , C. W. Yu , S. Y. Chen , C. L. Lin , and C. Y. Chuang , “Application of Surface‐Enhanced Raman Scattering in Rapid Detection of Dithiocarbamate Pesticide Residues in Foods,” Applied Surface Science 558 (2021): 149740.

[22] C. J. Rusin . Synthesis and Application of Colloidal Substrates for In‐Solution Surface Enhanced Raman Scattering (University of Alberta, 2020).

[23] P. Ma , L. Wang , L. Xu , J. Li , X. Zhang , and H. Chen , “Rapid Quantitative Determination of Chlorpyrifos Pesticide Residues in Tomatoes by Surface‐Enhanced Raman Spectroscopy,” European Food Research and Technology 246 (2020): 239–251.

[24] X. Wang , S. Ai , A. Xiong , et al., “SERS Combined With QuEChERS Using NBC and Fe_3_O_4_ MNPs as Cleanup Agents to Rapidly and Reliably Detect Chlorpyrifos Pesticide in Citrus,” Analytical Methods 15, no. 45 (2023): 6266–6274. 37955430 10.1039/d3ay01604h

[25] C. D. Albuquerque and R. J. Poppi , “Detection of Malathion in Food Peels by Surface‐Enhanced Raman Imaging Spectroscopy and Multivariate Curve Resolution,” Analytica Chimica Acta 879 (2015): 24–33. 26002473 10.1016/j.aca.2015.04.019

[26] (EFSA) EFSA , L. Carrasco Cabrera and P. Medina Pastor , “The 2020 European Union Report on Pesticide Residues in Food,” EFSA Journal 20, no. 3 (2022): e07215. 35386573 10.2903/j.efsa.2022.7215 PMC8965801

[27] G. T. Reddy , M. P. K. Reddy , K. Lakshmanna , et al., “Analysis of Dimensionality Reduction Techniques on Big Data,” IEEE Access 8 (2020): 54776–54788.

[28] I. Nitze , U. Schulthess , and H. Asche , “Comparison of Machine Learning Algorithms Random Forest, Artificial Neural Network and Support Vector Machine to Maximum Likelihood for Supervised Crop Type Classification,” in Proceedings of the 4th GEOBIA, Rio de Janeiro, Brazil (INPE ‐ Brazilian National Institute for Space Research, 2012): 7–9.

[29] R. Brasca , H. C. Goicoechea , and M. J. Culzoni , Multiway Calibration Approaches for Quality Control of Food Samples (Elsevier, 2018).

[30] S. P. Adam , S. A. N. Alexandropoulos , P. M. Pardalos , and M. N. Vrahatis , “No Free Lunch Theorem: A Review,” in Approximation and Optimization: Algorithms, Complexity and Applications, ed. I. Demetriou and P. Pardalos (Springer, 2019), 57–82.

